# Premarital sexual practice and its predictors among university students: institution based cross sectional study

**DOI:** 10.11604/pamj.2017.28.234.12125

**Published:** 2017-11-15

**Authors:** Mohammed Akibu, Fiseha Gebresellasie, Fitsum Zekarias, Wintana Tsegaye

**Affiliations:** 1 Department of Midwifery, Institute of Medicine and Health Sciences, Debre Berhan University, Debre Berhan, Ethiopia; 2 Korem Health Center, Korem Town, South Tigray, Ethiopia; 3 Department of Public Health, Institute of Medicine and Health Science, Debre Berhan University, Ethiopia; 4 Concordia University, Montreal, Qubec, Canada

**Keywords:** Predictors, premarital sexual practice, sexual debut, university students

## Abstract

**Introduction:**

Adolescents are susceptible to different social, peer and cultural pressures that Drives them into earlier sexual experimentation. Despite the fact that delaying sexual activity until marriage reduces the spread of HIV/AIDS and various sexually transmitted infections (STI), sexual activities among youths have been reported to be increasing worldwide.

**Methods:**

Institution Based cross-sectional survey was conducted between January 2016 to March 2016. A total of 604 students were included in the study using multi-stage sampling technique. Mixed Quantitative and Qualitative approach was applied. Bivariate and multivariate logistic regression model was fitted to identify factors associated with premarital sexual practice.

**Results:**

The proportion of premarital sexual practice in the study area was found to be 54.3%. The mean age of first sexual debut was 18.7±1.96. Half (50.6%) of these sexual activities were performed because of student's interest to meet their sexual desire. Being Male, watching pornography and High academic performance were the factors significantly associated with premarital sexual practice.

**Conclusion:**

The study had revealed that more than half of the participants were sexually active. Being male, watching pornography and high academic performance were predictors of premarital sexual practice. Therefore, Institute of Medicine and health science, gender office and HIV resource center have to organize different programs targeting at bringing behavioral change to minimize the prevailing prevalence of premarital sexual practice as well as its common consequences.

## Introduction

Adolescent stage of human development is an important phase of sexual maturation. During this period, adolescents often become active psychologically and sexually. That is due to physiological changes, peer pressure and their tendency of resisting to behavioral change through different forms of denial and rationalism [1]. These people are susceptible to different social, peer and cultural pressures that may lead them to earlier sexual experimentation [2]. Despite the fact that delaying sexual activity until marriage reduces the spread of HIV/AIDS and various Sexually transmitted infections (STI), sexual activities among youths have been reported to be increasing worldwide [3, 4]. Based on the report from Joint united nation program (UNAIDS) on HIV/AIDS in 2008, people aged 15-45 account for 42% of new HIV infection and 80% of these are from Sub-Saharan countries [5]. Similarly,16 million girls aged 15 to 19 get pregnant each year globally and 3 million of these pregnancies end up with unsafe abortion [6]. In spite of this, different studies in Sub- Saharan Africa revealed high and escalating level of premarital sexual activities among adolescents [4]. Students attending higher institutions often ignore their sexual and reproductive health and engage in risky sexual behavior because of the liberal nature of their university stay [7, 8]. Studies showed that the proportion of students engaging in pre-marital sexual practice reached 60.9 % in Addis Ababa [9]. Other studies have revealed 42.7% and 28.3 % prevalence of earlier sexual debut in Ethiopian higher institutions [10, 11]. Unless proper institution and age targeted interventions has been launched, such behavior can put students at greater risk of different health problems. However, little has been documented about premarital sexual activities and related risky sexual behavior in the study area. Therefore, this stud was intended to identify the magnitude of premarital sexual practice and associated factor among students of Debre Berhan University (DBU).

## Methods

**Study area and period:** The study was conducted at Debre Berhan University (DBU), which is one of governmental academic institutions in Ethiopia. DBU is located in Amhara regional state, northshowa zone, Debre berhan town, which is 130 km away from Addis Ababa in the North east. The university incorporates 8 colleges and has 33 streams. The total number of DBU regular students was 10,551, out of this 62.48% are males and the rest 37.52% are females (Debre Berhan university, office of registrar, unpublished). The study was Conducted from January-March 2016.

**Study design:** Institution based cross sectional survey was employed among randomly selected regular students of Debre Berhan University.

**Sample size determination and sampling procedure:** Single population proportion formula was used to calculate a sample size, by using 60.9% expected magnitude of pre-marital sexual practice [9]. Design effect of 1.5 and 15% Non-response rate were considered. Multi-stage sampling technique was employed to select study participants. There were eight colleges in the university. Initially, simple random sampling was applied to select three representative colleges. Then, the calculated sample size (n= 620) was proportionally allocated to each of selected colleges. Next, additional Lottery method was applied to identify two departments from each selected college's. Finally, systematic random sampling taking Kth value of 5 was used in order to identify the study participants from all representative departments. Students' ID number was taken as a sampling frame.

### Operational definition

**premarital sexual practice:** Penetrative penile or vaginal intercourse at least once before formal marriage.

**Khat chewer:** Khat is an ever; green stimulant plant that is extensively cultivated in highlands of Ethiopia. A student was considered a chewer if she/he answered yes to the question “have you chewed Khat previously“.

**Consistent use of condom:** regular condom use in every sexual activity.

**Data collection and quality control:** Quantitative data were collected using pre-tested and self-administered questionnaire at the participant's class room. The questionnaire was tested for internal consistency (reliability) by Cronbach´s Alpha test using statistical package for social sciences version (SPSS) 20.0. Similarly, content validity was cross-checked by a Reproductive health expert. Qualitative Data were collected using semi-structured interview guide checklist. Focus group discussion (FGD) among two group of students was carried out. Each group was made up of eight discussants.

**Data processing and analysis:** The quantitative data were coded, cleaned and entered into EPI-INFO version 3.5.3 and it was exported to SPSS version 20 for statistical analysis. Binary and multiple logistic regressions was done to examine the possible association between the determinant and outcome variable. In multiple logistic regression variables with P value <0.05 were considered statistically significant. The qualitative data were recorded using tape recorder and the data were transcribed to English by experts and the analysis was carried out using NVivo 7 and the generated result were triangulated with the quantitative results.

**Ethical consideration:** Ethical approval was obtained from the Institutional Review Board (IRB) of Debre Berhan university, Institute of Medicine and health science. Written and verbal consent were taken from the study participants. Information's obtained were kept confidential and anonymous.

## Results

**Socio-demographic characteristics:** A total of 604 students took a part in the study making a response rate of 96%. The age of the respondents ranged from 18 to 25 with a mean age of 22.03 ±1.9. More than half 316 (52.3%) of participants were Males. About 461 (76.3%) of the respondents were Orthodox religion followers. Nearly one third 200 (33.1%) participants had Illiterate mothers, whereas 248 (41.1%) of their fathers had atleast attended primary education. Half 306 (50.7%) of respondents were from rural parts of the country. Majority 332 (55%) of participants were middle level achievers on their field of study (Table 1).

**Table 1:** Socio-demographic characteristics of regular undergraduate students of Debre Berhan University, 2016

Socio-demographic characteristics	Frequency	Percentage
**Age**		
18 - 21	235	38.9
22 – 25	369	61.1
**Sex**		
Male	316	52.3
Female	288	47.7
Religion		
Orthodox	461	76.3
Muslim	57	9.4
Catholic	39	6.8
Protestant	47	7.8
**Mother educational status**		
Illiterate	200	33.1
Primary education	202	33.4
Secondary education	105	17.4
Higher education	97	16.1
**Father Educational status**		
Illiterate	116	19.2
Primary education	248	41.1
Secondary education	104	17.2
Higher education	136	22.5
**Academic performance**		
Lowe Achiever	107	17.7
Medium Achiever	332	55
High Achiever	165	27.3
**Place of residence**		
Urban	298	49.3
Rural	306	50.7

**Premarital sexual practice:** The proportion of premarital sexual practice in the study area was 328 (54.3%). The mean age of first sexual debut was 18.74 SD ±1.96. More males 182 (55.5%) than females 146 (44.5%) were involved in earlier sexual debut. The mean age of first sexual practice was 17.8± 1.59 and 18.4±1.9 for Males and Females respectively. Half 166 (50.6%) of participants were Involved in premarital sexual practice because of their desire for sexual intercourse (Table 2).

**Table 2:** Practice of premarital sexual intercourse among regular undergraduate students of Debre Berhan University, 2016

Variables	Frequency	Percentage
**Premarital sexual practice (n=604)**		
Yes	328	54.3
No	276	45.7
**Premarital sexual debut by sex (n =328)**		
Male	182	55.5
Female	146	44.5
**Reason for sexual Debut (n=328)**		
Desire for sexual activity	166	50.6
Fell in love	53	16.2
Casual	55	16.8
Peer pressure	42	12.8
Economic need	12	3.7

**Risky sexual behaviors:** Two third 234 (71%) of study participants had protected sexual intercourse in the last 12 months before the study. However, only 93(39.7%) of participants have used the condom consistently. The proportion of students who had multiple sexual partner were 70 (21.4%). Little above half 318 (52.6%) of participants reported that they watched pornographic films. 209 (34.6%) of students revealed alcohol intake in the last 12 months preceding this survey, of this, 72 (34.4%) claimed they are regular consumers. One in five students 123 (20.4%) reported chewing Khat in the last 12 months prior to this survey. Of these, 38 (30.9%) of them were regular Khat users (Table 3).

**Table 3:** Distribution of Risky sexual practices among regular undergraduate students Debre berhan university, 2016

Risky sexual behaviors	Frequency	Percentage
**Condom use (n=328)**		
Yes	234	71.3
No	94	28.7
**Frequency of condom use (n=234)**		
Always	93	39.7
Occasionally	141	60.3
**Number of sexual partners (n=328)**		
Only one	258	78.6
Two or more	70	21.4
**Pornography (n=328)**		
Yes	319	52.8
No	285	47.2
**Alcohol (n=328)**		
Yes	209	34.6
No	395	65.4
**Frequency of consumption (n=209)**		
Usual consumer	46	22.1
Moderate consumer	72	34.4
Occasional consumer	91	43.5
**Khat (n=328)**		
Yes	123	20.4
No	481	79.6
**Frequency of chewing (n=123)**		
Usual chewer	38	30.8
Moderate chewer	58	47.1
Occasional chewer	27	21.95

**Factors associated with premarital sexual practice:** In Multivariate logistic regression, Males were 2 times more likely to engage in premarital sexual debut than females (AOR 2.3 95% CI = 1.59-3.3). Watching pornographic films was another Predictor of premarital sexual practice, as students watching such films had 2.3 times higher tendency of practicing pre-marital sexual intercourse (AOR 2.3 95% CI = 1.6-3.27). Having higher academic performance was found to be a protective factor against pre-marital sexual practice (AOR 0.43 95% CI = 0.25-0.74). Students with high academic performance were 57% less likely to engage in premarital sexual debut than lower rank students (Table 4).

**Table 4:** Bivariate and Multivariate analysis of factors associated with Premarital sexual practice among regular undergraduate students of Debre Berhan university, 2016

Variables	Earlier sexual practice		COR (95% CI)	AOR (95% CI)
	Yes	No		
**Sex**				
Male	194	122	1.82 (1.32-2.52)	2.3(1.59-3.3)^++^
Female	154	134	1	1
**Permanent residence**				
Urban	176	122	1.46 (1.06-2.016)	1.2(0.8-1.75)
rural	152	154	1	1
**Academic performance**				
High	80	85	0.53 (0.32-0.86)	0.43 (.25-.74)^++^
Medium	177	151	0.66 (0.42-1.03)	0.57 (0.35-0.91)
Low	71	40	1	1
**Pocket money**				
No income	40	44	1	1
Up to 300	130	122	1.17(0.75-1.9)	0.94 (0.55-1.56)
300 to 500	86	60	1.58 (0.92-2.7)	1.1(0.6-2.0)
500 to 1000	67	44	1.67(0.94-2.97)	1.5(0.76-.29)
More than 1000	5	6	0.917(0.26-3.24)	1.025 (0.25-4.13)
**Pornography**				
Yes	207	112	2.5(1.8-3.4)	2.3 (1.6-3.27)^++^
No	121	164	1	1
**Mother Education**				
Illiterate	100	100	1	1
Primary education	103	99	1.04(.704-1.54)	0.98(0.64-1.5)
Secondary education	71	34	2.09 (1.27-3.4)	1.5(0.84-2.66)
Higher education	54	43	1.26(.77-2.04)	0.92(5.11-1.65)

^++^Statistically significant p value <0 .05

### Qualitative result

**Premarital sexual practice and risky sexual behaviors:** According to the reflection of discussants, premarital sexual practice was very common in the study area and It's regarded as the basic element of relationship at university level. One of the discussant said that:

"When you begin relationship, the first thing you want to test is the sexual compatibility between you and your pair. Either of them could not refuse to engage in sexual activity after dating, if so, they will get separated. Here in campus you do have a lot of options (girls for boy, boys for girl), therefore you won't keep wasting your time with someone who doesn't like to give himself to you"

They further explained that the freedom (being away from their family) students had at this level of their development plays a major role in initiation of sexual intercourse before formal marriage. One of the discussants said:

"To your surprise, most of females and even some males have never had sex before they get here, but once they stayed in campus for some time and enjoyed the freedom they never had with their family, the first thing they want to do is spending times out of campus, going night clubs and establishing sexual relationship that doesn't even last for a few monthes"

The other notion mentioned was, girls want to achieve a good financial status to satisfy their needs. Therefore, in most of cases sleeping with older peoples (sugar daddies) will be their preferred way. Family support will not be sufficient enough to meet the demand of students other than school stuffs. One of the discussant said

"It's now becoming fashion to sleep with someone who is rich and generate money. There are student brokers who recruit girls and present them to those who can pay back big money. Most girls find the amount of money and properties offered by these people irresistible. I know some girls personally who were completely smart (Well behaving) but eventually trapped in this activity".

Most discussants agreed that pornographic movies play a significant role for initiation of earlier sexual practice and these films are the primary reference materials for students to improve their sexual performance. One of the discussant said

"I think there is no doubt about the power these movies have. Most students here rely on pornographic movies to master better sexual practice and learn different sex positions. Particularly boys are very concerned to learn about how to take their girls to orgasm very quickly and they feel porn films are the best approach to follow. You would have been amazed if you go and see how many students are busy in downloading these movies".

## Discussion

The study investigated practice of premarital sexual intercourse and its determinants among unmarried regular students of Debre Berhan university. The overall prevalence of premarital sexual practice in the study area was 54.3%. This finding was higher than the result obtained from, madawalabu, wollega and Bahir Dar University [10-12]. However, the result was lower than the report from Alkan University College, Addis Ababa, Mbarara University of Science and Technology, Uganda and university students in Madagascar [9, 13, 14]. This disparity could be justified by the difference in background of the study participants, variation in study area and sample size and cultural Dissimilarity between countries. The age at which sexual debut was performed determine student's vulnerability for various unpleasant consequences including STI and unwanted pregnancy. The mean age of first sexual intercourse in this report was 18.7. This finding was comparable with the report from bahir dar university, among university students in Madagascar and national report by EDHS [12, 14, 15]. This study revealed that 70 (21.4%) of participants had Multiple sexual partners. This report was lower than the finding from Madawalabu university, bahirdar university and Wolayta sodo university [10, 12, 16]. On contrary, the result was comparable with findings of Jimma university and Haramaya university [17, 18]. This could be due to the time gap between these studies, in that; there will be different interventions through time targeted at improving adolescents' risky sexual behaviors.

The proportion of sexual intercourse protected with condom use was 71.3%. This result was higher compared to reports from Ghana and Kenya which was 50% and 42% respectively [19, 20]. This could be due to variation in participants' awareness about risk of such activity, time gap between these studies and difference in study area. However, consistent use of condom was lower than the overall all condom utilization, in that only 93 (39.7%) of were used condom always. This figure was compatible with finding by Wondemagegn M and Certain MD et al [12, 21]. Conversely, it was much lower compared to results from Alkan university college and Mahidol university Thailand [9, 22]. This is an Input for stakeholders to sustain their effort on advocating regular condom use. Being male was the only socio-demographic variable associated with premarital sexual intercourse. Males were two times more likely to engage in premarital sexual debut than females. This finding was in line with the report from Nekemte town, among adolescents in Malaysia and Oromia, South west, Ethiopia [23-25]. This could be due to the cultural and social values and freedom given to males in certain societies. The cultural norms that advocate keeping girls' virginity until their marriage could also be another explanation. Watching pornography was another factor determining practice of premarital sexual intercourse in the study area. Students who were watching pornography were more likely to perform earlier sexual practice. This figure was consistent with findings from North East Ethiopia, west Gojjam zone, North Western Ethiopia and a study in Asian country [26-28]. This might be due to the potential stimulating effect on sexual desire and its power to increase perceived pleasure from sexual intercourse. Another important predictor of premarital sexual intercourse was students' level of academic performance. This study revealed that students who were higher academic achievers were refrained from such sort of activity than low achiever ones. This finding was similar to the report from eastern Ethiopia [29]. This might be due to the belief that top ranking students are quite busy with academic activities because of greater desire to maintain their high performance, whereas low achievers are dependent upon others, therefore, have a lot of free time to expose themselves to different risky sexual behaviors which eventually lead to sexual experimentation.

## Conclusion

In summary, the study has revealed that more than half of the participants were sexually active. Being male, watching pornography and High academic performance were factors associated with practice of premarital sexual intercourse. Therefore, Institute of Medicine and health's science, gender office and HIV resource center has to organize different programs targeted at bringing behavioral change to minimize the prevailing prevalence of premarital sexual practice as well as its common consequences.

### What is known about this topic

- A study by Bekana Fekecha in Ethiopia and Mee-lian Wong in Asia, reported that pornographic movies were a strong predictor of premarital sexual practice;
- Tomas Benti in Madawalabu University and Lee Lk in Malaysia revealed that males were more active to engage in premarital sexual practice than females.

### What this study adds

- The study has found that high academic performance was a protective factor against premarital sexual practice;
- According to the finding of this study, alcohol and chat intake had no significant association with earlier sexual debut.

## Competing interests

The authors declare no competing interests.
